# Two-pore channels: going with the flows

**DOI:** 10.1042/BST20220229

**Published:** 2022-08-12

**Authors:** Anthony J. Morgan, Lora L. Martucci, Lianne C. Davis, Antony Galione

**Affiliations:** Department of Pharmacology, University of Oxford, Mansfield Road, Oxford OX1 3QT, U.K.

**Keywords:** Ca^2+^, lysosomes, NAADP, TPC

## Abstract

In recent years, our understanding of the structure, mechanisms and functions of the endo-lysosomal TPC (two-pore channel) family have grown apace. Gated by the second messengers, NAADP and PI(3,5)P_2_, TPCs are an integral part of fundamental signal-transduction pathways, but their array and plasticity of cation conductances (Na^+^, Ca^2+^, H^+^) allow them to variously signal electrically, osmotically or chemically. Their relative tissue- and organelle-selective distribution, together with agonist-selective ion permeabilities provides a rich palette from which extracellular stimuli can choose. TPCs are emerging as mediators of immunity, cancer, metabolism, viral infectivity and neurodegeneration as this short review attests.

## Acidic Ca^2+^-stores

These H^+^-rich (acidic) vesicles encompass a spectrum of organelles that include endo-lysosomes, lysosome-related organelles and secretory vesicles which are endowed with the common ability to store and release Ca^2+^. That is, in addition to their roles of trafficking cargo, repairing membranes, degrading macromolecules and nutrient sensing, acidic vesicles generate Ca^2+^ signals. The purpose of this article is to update our previous overview [1] with more recent developments pertaining to one particular family of Ca^2+^-permeable channels found on such acidic vesicles — the TPCs (two-pore channels) — and we confine our remarks to the mammalian channels.

**Figure 1.**
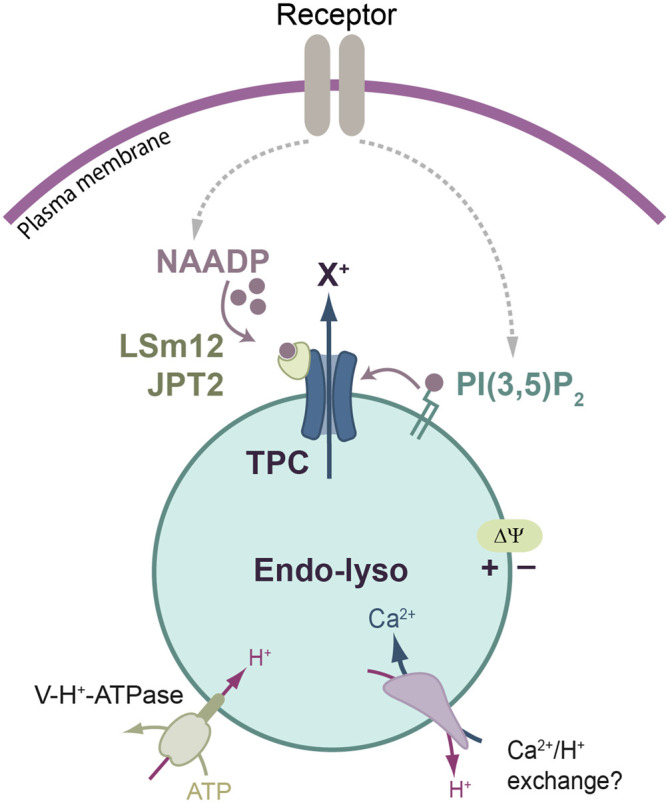
Second-messenger synthesis couples plasmalemmal receptors to endo-lysosomal TPCs. A model whereby cell-surface receptors can promote the synthesis of either second messenger, the dinucleotide, NAADP or lysosome-specific lipid, PI(3,5)P_2_. Cations (X^+^) exit the acidic vesicle when TPCs are gated by NAADP via small accessory proteins, LSm12 or JPT2, or by PI(3,5)P_2_ that binds directly to TPCs. The lysosomal lumen is acidic by virtue of the V-H^+^-ATPase, which is also the primary drive of the luminally positive membrane potential (ΔΨ). In one model, the H^+^ gradient drives Ca^2+^ uptake via an unknown exchanger.

The free [Ca^2+^] within endosomes is tens of micromolar, whereas in lysosomes it is ∼300–600 µM [2,3] and comparable to that of the other major Ca^2+^ store, the endoplasmic reticulum (ER). The route of lysosomal Ca^2+^-filling remains unclear with candidates being either Ca^2+^/H^+^ exchange [2,4] (Figure 1), Ca^2+^ transfer from the ER via IP_3_ receptors (IP_3_Rs) [5] or the ATP13A2 transporter [3]. Whilst cytosolic [Na^+^] is ∼12 mM, the lysosomal luminal [Na^+^] is reported from 21 mM [6] to 150 mM [7], though 21 mM may be more reliable since low temperatures and the lack of ATP (in [7]) disrupt normal monovalent cation gradients [8,9]. The resting membrane potential (ΔΨ) across lysosomes is luminally positive (19–100 mV) [2,10,11] (Figure 1).

Two features distinguish the acidic Ca^2+^ stores from the ER: first, they are a diminutive Ca^2+^ source compared with the ER (typically 10% of the ER volume [12]) and therefore, the total amount of Ca^2+^ that is released by acidic Ca^2+^ stores is small by comparison with the ER's; second, acidic vesicles are arguably more motile, traversing large distances relative to their size. Both these features afford acidic Ca^2+^ stores the capability of substantially impacting physiology and ‘punching above their weight’.

## TPCs — structure and distribution

**Figure 2.**
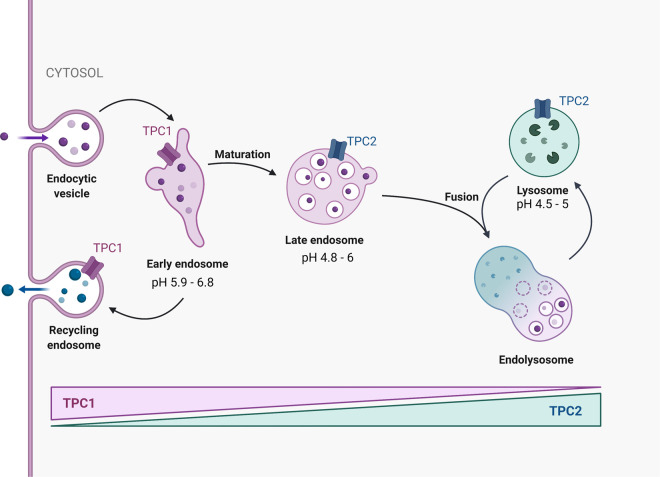
Distribution of TPC channels throughout the endo-lysosomal system. TPC1 channels are predominantly found in less acidic, earlier compartments, whereas TPC2 is found in later, more acidic vesicles. Trafficking of cargo through the endo-lysosomal system (e.g. endocytosis, viruses, bacterial toxins) as well as vesicle movement and fusion is also subject to TPC control.

Analogous to IP_3_Rs evoking Ca^2+^ release from the ER, the gating of Ca^2+^-permeable channels on acidic vesicles increase cytosolic Ca^2+^. There are multiple families of channels found across the vesicular continuum and include the TPCs, mucolipins (TRPMLs) and P_2_X4 receptors. Their pattern of expression is not only cell-type dependent but also aligned with certain vesicle populations. Mouse and human each contain two isoforms, TPC1 and TPC2, that are ubiquitously expressed throughout the body and particularly high in kidney and immune cells [2]. Generally, TPC1 predominates in mildly acidic vesicles (recycling endosomes, early endosomes; pH 5.7–6.9) whilst TPC2 is mainly found in more acidic late-endosomes/lysosomes or secretory vesicles (pH 4.0–5.6) [2] (Figure 2).

**Figure 3.**
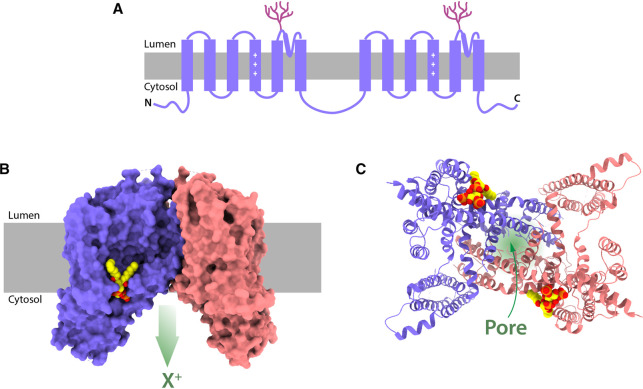
Structure of TPCs. (**A**) Topology cartoon of a TPC monomer with tandem repeats of two ‘Shaker’ domains (six transmembrane domains each). The positively charged amino acids (+++) in the S4 domains confer voltage sensitivity in TPC1. Magenta branches depict luminal glycosylation. (**B**) Cryo-EM structure of the human TPC2 dimer (PDB: 6NQ0) with the surface structure of A and B chains in blue and pink, respectively. The lipid, PI(3,5)P_2_, is shown as a space-filling model (yellow, red and orange) bound to a pocket in the A chain (a second lipid molecule, bound to the equivalent pocket on the B chain, cannot be seen behind). X^+^ represents the direction of cation flow. (**C**) human TPC2 as a ribbon diagram flipped 90° compared with (**B**) and viewed end-on from the cytosolic face. The central pore (green shading) is contributed to by both monomers. Both PI(3,5)P_2_ molecules bound are visible. Structures were generated using UCSF Chimera X [132].

Hot on the heels of the plant TPC atomic structure [13], mammalian TPC1 [14] and TPC2 [15] were resolved by cryogenic electron microscopy and complemented recently by a zebrafish TPC3 structure [16]. The structure of all three isoforms is a similar TPC dimer that strikingly resembles voltage-gated ion channels, to which TPCs are evolutionarily related [17] (Figure 3). However, mammalian TPCs are unusual in the cation-channel pantheon in having a selectivity filter without charged residues [18] so that, instead, the main barrier to permeation is a steric hydrophobic gate that is relieved upon ligand binding [19]. Expression of the pore-forming region alone results in a constitutively active cation channel [20]. Unlike TPC2, TPC1 is voltage-dependent by virtue of a unique voltage-sensing S4 domain [14] (Figure 3).

## Channel regulation

As important signalling switches, Ca^2+^ channels exhibit sensitivity to multiple inputs, be they ions, ligands or proteins, and TPCs are modulated by all three classes.

### Ca^2+^ feedback

Classically, IP_3_Rs are regulated strongly by both cytosolic and luminal Ca^2+^ which amplify or dampen signals [21]. The Ca^2+^ effects upon TPCs are, however, more variably reported. On the one hand, TPC1 was stimulated by cytosolic Ca^2+^ [22] via a shift in its voltage activation [23] which might reinforce endosomal Ca^2+^ release. On the other hand, others do not find any effect of cytosolic Ca^2+^ on TPC1 [24]. An effect of cytosolic Ca^2+^ on TPC2 is not reported, and its vestigial cytosolic EF-hands lack the residues for Ca^2+^-binding [15]. Regulation by luminal Ca^2+^ is equally inconsistent. Luminal Ca^2+^ has been shown to have no effect on TPC1 [22], an inhibitory one (by locking into a closed state) [23] or a stimulatory one [25]. For the other isoform, TPC2 is activated by luminal Ca^2+^ [26]. The reason for the discrepancies is unknown.

### Second messengers

Unlike monogamous IP_3_Rs, TPCs are more promiscuous and respond to either of two second-messengers. One is the cytosolic soluble second messenger, NAADP (nicotinic acid adenine dinucleotide phosphate), the other is a lysosome-specific lipid, PI(3,5)P_2_ (phosphatidylinositol 3,5-bisphosphate) (Figure 1). Befitting a messenger role, their levels increase in response to different cell stimuli e.g. [27–29], and TPC-dependent Ca^2+^ signals (or currents) can be evoked by either ligand. Whilst the route of PI(3,5)P_2_ synthesis is clear (PIKfyve [30]), that for NAADP has, historically, been uncertain. Candidates include CD38 (or related ADP-ribosyl cyclases) [31–33], SARM1 [34] and, more recently, the NADPH oxidases, DUOX1/2 [35].

Thanks to the atomic structures, we know that PI(3,5)P_2_ binds directly to TPC1 and TPC2 (Figure 3) and how this gates the channel [14,15]; mutagenesis of complementary basic amino acids in the binding pocket abolishes activation by the phospholipid [14,15,36]. For NAADP, TPC stimulation is indirect, with NAADP binding to a smaller, accessory protein(s) (Figure 1). Recent screens have finally identified NAADP-binding proteins that mediate the gating of TPC, namely LSm12 [37] and JPT2 [38] (note: JPT2/HN1L was also reported to activate ryanodine receptors (RyRs) [39]). These proteins were unexpected candidates given that LSm12 is an RNA-binding protein and JPT2 is otherwise mechanistically orphaned (although linked to cancers) [40]. Attesting to its importance, LSm12 deletion is embryonic lethal [37]. Where and how these proteins bind to TPCs (and whether there is any isoform selectivity [40]) will prove a key future direction.

The potential for two molecular messengers to converge upon one channel is unusual, and the physiological consequence of this duality is ill-defined. Whether either (or both) messengers is required for TPC activation requires further work and may also be context-sensitive. For example, in the same macrophage, TPC2 responds to PI(3,5)P_2_ for macropinosome resolution (i.e. shrinkage and resorption) [41], but to NAADP for TPC-dependent phagocytosis [42]. Moreover, inhibition of PI(3,5)P_2_ synthesis with vacuolin-1 [43] did not alter NAADP-induced Ca^2+^ release [44] implying there is little interaction between the messengers, at least in fibroblasts. The messengers’ kinetics, uniqueness, redundancy or potential synergy may shape the signalling palette from which stimuli can choose.

### Ion permeabilities

Ionic permeabilities inform us as to the possible (and multiple) roles of TPCs. For mammalian TPC1, its permeability sequence has been reported as Na^+ ^> K^+ ^> Ca^2+^ [24] or H^+ ^> K^+ ^> Na^+^ ≥ Ca^2+^ [22], whereas for TPC2 its rank order has been given as Na^+ ^> Ca^2+ ^> K^+ ^> Cs^+^ [7,26,44–47]. In spite of common trends, the absolute permeability ratios recorded for a given TPC isoform perplexingly vary, e.g. the P_Ca_/P_Na_ for TPC1 is recorded as 0.98 [22], ∼0.05 [23] and 0.005 [24]. As with the Ca^2+^ feedback above, the different methodologies (e.g. lipid bilayers, whole-lysosome recording, ectopic expression in plant vacuoles) may be a contributing factor to some of the discrepancies.

Just as the plasmalemmal NMDA receptor is a transducer of both electrical (Na^+^) and chemical signals (Ca^2+^), so too may TPCs be multi-functional and alter endo-lysosomal ΔΨ and osmolarity (Na^+^), cytosolic Ca^2+^ signals or vesicular pH (pH_L_) (Figure 5). Note that egress of Na^+^ depolarises the endo-lysosomal membrane [48], which impacts both Ca^2+^-refilling and -release [2,49,50], and the ability of the electrogenic V-H^+^-ATPase to acidify the lumen [51]. Acidic vesicle ΔΨ is manifestly important physiologically e.g. for vesicular fusion [52], cholesterol storage [49] and phagocytosis [50].

When a Na^+^ conductor, TPC1 modulates vesicular ΔΨ and electrical excitability [24]. In an osmotic modality, TPC1/TPC2 co-ordinate macropinosome resolution when their Na^+^ fluxes drive Cl^−^ co-transport, water movement and pinosome shrinkage [41,53]. As Ca^2+^-permeable channels, TPCs have arguably garnered more attention physiologically (see below). Experimentally, it is currently not trivial to distinguish between the Na^+^ and Ca^2+^ modalities of TPC signalling in driving biological processes, in part due to our inability to monitor Na^+^ fluxes *in situ*.

**Figure 4.**
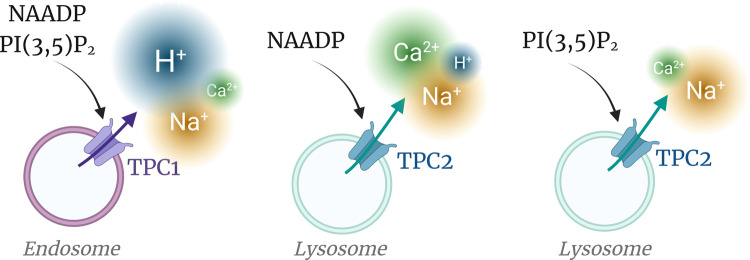
Ionic permeabilities are a function of the TPC isoform and stimulus. Models depicting the relative permeabilities to Ca^2+^, Na^+^ and H^+^ are conveyed by the size of the coloured plumes. For TPC2, soluble NAADP evokes TPC2 currents with comparable Ca^2+^ and Na^+^ conductances, whereas the lipid PI(3,5)P_2_ stimulates Na^+^-selective currents.

However, the permeability sequence of TPCs is not immutable and depends on the stimulating messenger. Activation of TPC2 via the PI(3,5)P_2_-pathway promotes a predominantly Na^+^ current (P_Ca_/P_Na_ ∼ 0.08), whereas the NAADP pathway evokes an eight-fold larger Ca^2+^ conductance (P_Ca_/P_Na_ ∼ 0.65) [54] (Figure 4). TPC1 may also exhibit ligand-dependent permeability, albeit more modestly, with PI(3,5)P_2_ shifting the P_Ca_/P_Na_ from 0.98 to 0.42 [22]. Ligand-induced permeability changes are a unique feature of TPCs and thereby resolve early controversies as to the permeant ions. Thus, by the judicious selection of messenger, TPCs may be recruited to signal via Na^+^ (osmolarity, ΔΨ) or Ca^2+^ or pH_L_.

**Figure 5.**
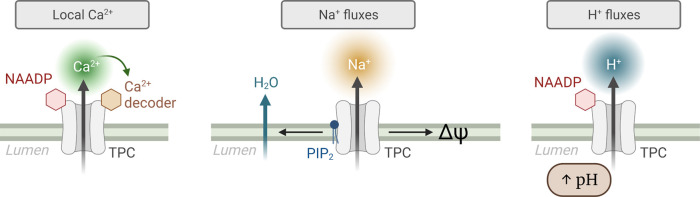
Models of different ionic signalling modalities for TPC2. With NAADP as the messenger, it binds to its accessory protein (LSm12 or JPT2, red hexagons) to evoke local Ca^2+^ nanodomains that are uniquely sensed by closely associated Ca^2+^-binding proteins (‘decoders’, brown hexagon)). When PI(3,5)P_2_ is the stimulus, Na^+^-selective currents are evoked which can depolarise the lysosome (ΔΨ) or promote osmotic changes and vesicle shrinkage (by Cl^−^ co-transport and concomitant water loss). NAADP can also promote H^+^ efflux through TPC2 and increase the lysosomal luminal pH (pH_L_).

How NAADP elevated the pH_L_ of acidic Ca^2+^ stores was unclear [29,55] until the demonstrations that both TPC1 and TPC2 conduct H^+^, i.e. efflux pathways from vesicles [22,54] (Figure 5). Therefore, NAADP may signal not just by an increase in cytosolic Ca^2+^, but by a coincident alkalinization of endo-lysosomes. Interestingly, pH_L_ changes parallel the Ca^2+^ signals in that H^+^ fluxes are stimulated by the NAADP- but not the PI(3,5)P_2_-pathway with TPC2 [54]. In part via effects on vesicular pH, TPC2 influences melanosome pigmentation [47,56] and autophagy [57].

In summary, different messengers evoke different ionic signals. This may explain, for example, why PI(3,5)P_2_ is selected for macropinosome resolution: the lipid favourably stimulates fluxes of Na^+^ (but not H^+^) to drive Cl^−^ and water loss [41]; NAADP would have been unfavourable since it evokes smaller Na^+^ fluxes and an increase in pH_L_ that could inhibit the essential Cl^−^ co-transport [53].

### Pharmacology

Compounds that modulate TPCs are a growing family, with currently more inhibitors to choose from than activators.

#### Inhibitors

Most of the inhibitors are pore-blockers. At high concentrations, traditional voltage-gated Ca^2+^-channel blockers interact with TPCs, e.g. verapamil inhibits TPC1 [54] and TPC2 [7] currents and NAADP-induced Ca^2+^ release [58,59]. Screens have re-purposed drugs as inhibitors of NAADP-induced Ca^2+^ release (by implication and by modelling, as TPC blockers) [60–62]. One broad-spectrum channel blocker, tetrandrine, is used to inhibit TPC2 [54,58,59] and TPC1 [63], and refinement of its structure has revealed more potent analogues towards TPC2, albeit with variable discrimination from TPC1 or TRPMLs [64]. The natural dietary flavonoid, naringenin, inhibits TPC1 and TPC2 with low affinity [65] and other Asian-plant flavonoids (pratensein and duartin) block TPC2 [66].

In terms of antagonising the messengers, the only cell-permeant NAADP antagonists that we have are BZ194 [67], the original Ned-19 [68], and its minimally modified analogue, Ned-K [69,70]. We do not yet have any specific inhibitors of the lipid-activation site, although high concentrations of Ned-19 unexpectedly block PI(3,5)P_2_-induced TPC2 currents [58].

#### Activators

Historically, activation of TPCs in intact-cell populations has been limited to NAADP delivery via liposomes [71,72] or cell-permeant NAADP (NAADP/AM) [73] which is notoriously labile. The recent discovery of stable, cell-permeant agonists that mimic these two TPC activators will open up the field, even if robust Ca^2+^ responses require the ectopic expression of TPC2 [54]. Each mimetic targets the TPC2 isoform (TPC2-A1-N [NAADP mimetic] and TPC2-A1-P [PI(3,5)P_2_ mimetic]), and are selective for TPC2 over TPC1 and TRPMLs [54]. TPC2-A1-P requires the lipid-binding site on TPC2 [54] (Figure 3) — likely a direct interaction with the channel — but the TPC2-A1-N activation mechanism is currently unclear. Does it bind to the NAADP accessory proteins LSm12/JPT2, or does it bind to TPC2 directly and mimic their interactions?

Interestingly, photo-release of another lipid, sphingosine, acutely evoked Ca^2+^ signals via TPC1 (but not TPC2) [74]; is this an underexplored new pathway? Surprisingly, tricyclic antidepressants (TCAs) and the motor-neuron-disease medication, riluzole, are TPC agonists [75], but their poly-pharmacology towards other transporters will probably limit their usefulness in intact cells.

Of broad interest, the mTOR inhibitor, rapamycin, evokes TPC2-dependent Ca^2+^ transients in myocytes [59] and promotes TPC2-mediated currents in synergy with PI(3,5)P_2_ [76]. Likewise, rapamycin activates TRPML1 by binding to the channel and synergises with the messenger, PI(3,5)P_2_ [77,78]. Whilst rapamycin activation of TRPML1 is direct, activation of TPC2 is suggested to be indirect via inhibition of mTOR [59,76,79].

### Protein regulators

Other signalling inputs may interact with TPCs including protein kinases such as LRKK [80], JNK/p38 [81], mTOR [79] and the small GTPase, Rab7 [82]. Protein kinase A was proposed to modulate TPC2 currents via phosphorylation of Ser666 [46] although, curiously, this residue lies within the lysosomal lumen and not accessible to cytosolic cAMP signals. In some cases, the physiological context for these modifiers is poorly defined.

### Polymorphisms

In the global population, TPC2 naturally occurs with a spectrum of different polymorphisms [83] and some impact TPC2 function. The degree of melanin pigmentation is inversely related to TPC2 activity [66] and two gain-of-function (GOF) polymorphisms (in different regions of the TPC2) each promoted blond-hair colour by independent mechanisms [76]. More recently, it was shown that the M484L mutation required an additional ‘permissive’ L564P polymorphism [83]. GOF mutants certainly produce larger currents in response to PI(3,5)P_2_ [54,76,83] or to the TPC2 agonists TPC2-A1-N and TPC2-A1-P [54]. However, it is less certain which signalling modality of TPC2 these polymorphisms produces the phenotype when roles for Ca^2+^ [56], Na^+^ (ΔΨ) [47,83] and pH [47,56,83] have all been posited for pigmentation.

## TPC Ca^2+^-decoding

How are TPC-dependent Ca^2+^ signals converted (decoded) into downstream responses? A common theme is that TPCs affect vesicle formation, trafficking, maturation and movement, many of which are sensitive to Ca^2+^ and lysosomal membrane potential. Being small Ca^2+^ stores, endo-lysosomes are designed to generate local rather than global Ca^2+^ signals, and TPCs couple to downstream physiology via local Ca^2+^ signals for which other Ca^2+^ sources cannot substitute, as exemplified by phagocytosis [41,42], exocytosis [84], membrane contact site (MCS) formation [85], receptor trafficking [86,87], development [31], vesicular fusion/motility [88,89]. Ca^2+^-decoders include Ca^2+^-dependent channels, protein kinases/phosphatases and membrane-fusion machinery.

Via Ca^2+^ release, TPCs trans-activate Ca^2+^-regulated ion channels on other membranes that are closely apposed, probably at MCSs. Via Ca^2+^-induced Ca^2+^ release (CICR), IP_3_Rs or RyRs on the ER can amplify the small Ca^2+^ release from endo-lysosomal TPCs to evoke global Ca^2+^ signals [90]. At the plasma membrane, Ca^2+^-sensitive channels (e.g. TRPM4/5 [91]) depolarise pancreatic β-cells following glucose-induced bursts of local NAADP/TPC Ca^2+^ signalling [91] under the plasma membrane [92]. Although there are also Ca^2+^-sensitive K^+^ channels on lysosomes that regulate vesicular ΔΨ, so far only Ca^2+^ released by TRPML1 has been linked to their activation [49].

How local TPC Ca^2+^ signals are otherwise decoded is underexplored. Privileged TPC-coupling to downstream processes implies that Ca^2+^-sensitive decoding proteins are intimately associated with TPCs and sense these high Ca^2+^ nanodomains. Several TPC interactomes have been published (reviewed in [93]), but surprisingly few Ca^2+^-binding proteins have been pulled out (e.g. annexins, although interactions have not always been validated). Phagocytosis is uniquely driven by local Ca^2+^ from TPCs (but not global Ca^2+^ signals) [42], where the Ca^2+^-dependent phosphatase, calcineurin, may be the Ca^2+^ decoder [42].

The molecular switches downstream of the immediate Ca^2+^-binding decoders are growing, and the GTPase, dynamin, has been linked to the NAADP/TPC axis during ‘inward’ trafficking at phagocytosis [42] and endocytosis of the glucose transporter, GLUT1 [94]. Regarding downstream phosphorylation, the MAP kinase, ERK1/2, mediates cell proliferation driven by TPC2 [95], although ERKs are not themselves Ca^2+^-binding proteins. In neuronal cells, the NAADP/TPC axis activates AMPK during autophagy [96]. In melanomas, TPC2 activates MITF (microphthalmia-associated transcription factor) via a GSK3β phosphorylation pathway [66]. Affirming a role in trafficking, fusion and vesicle motility, TPC1/2 interactomes are heavily biased towards SNARE complex proteins such as VAMPs and syntaxins [93].

## TPCs and health

Our appreciation of the importance of TPCs is expanding, but the following, recent examples incidentally reinforce that the precise molecular details of the circuitry are often lacking and we do not know the messenger, permeant ion or the decoders. Our understanding of the roles of TPCs is still in its infancy.

TPCs contribute to neuronal homeostasis [97]. The neurotransmitter, glutamate, uses an NAADP pathway to drive Ca^2+^ signals [98–100] which in turn can drive neuronal autophagy via TPC1/2 [96]. TPCs are important for memory and long-term potentiation [99,101], neuroprotection [98] and axonal/neurite extension [72,102]. Aberrant TPC signalling may contribute to neurodegeneration (see below).

In the vasculature, the role of TPCs is growing and, in particular, at vasculogenesis. The proliferation of endothelial precursors cells is dependent upon NAADP and TPC1 [72,103], and several studies implicate TPC2 in angiogenesis e.g. [65,104]. During embryogenesis, TPC1 and TPC2 promote different aspects of muscle development, namely myoseptal junction formation [105] and myogenesis, respectively [106]. Moreover, innervation of the muscle likewise relies on the NAADP/TPC2 axis [102].

Metabolically, the nutrient-sensing kinase complex of mTOR inhibits TPC2 which thereby responds to nutrient status [79]. Reciprocally, TPC2-KO enhances mTOR activity [107]. Manipulation of TPC1 expression reveals a potential link to glucose and fat metabolism [108], and the net surface expression of GLUT1 [94] and GLUT4 [108] glucose transporters are under the control of endosomal TPC1, probably by regulating endocytosis. Deletion of TPC2 in mice exacerbates the effects of a high-fat diet by reducing cholesterol/triglyceride clearance [87,109], although this does not translate into weight gain [109], partly due to enhanced insulin sensitivity in the absence of TPC2 [109].

TPCs are abundantly expressed in immune cells and are involved in often complex Ca^2+^ circuitry to regulate vesicular trafficking events in an immune context [110]. Extracellular particle clearance and fluid sampling during ‘inward’ trafficking events like phagocytosis [42] and macropinocytosis [41] in macrophages are mirrored by TPCs controlling ‘outward’ events like exocytotic secretion of histamine in mast cells [63], of cytolytic factors in cytotoxic T-cells [84] and the surface-presentation of chemokine signalling molecules [111]. Thus, TPCs may be invaluable during anaphylaxis, pathogen clearance by the innate immune system and T-cell clonal expansion.

## TPCs and disease

Given their physiological roles — particularly of membrane and protein trafficking — TPCs are implicated in a wide range of diseases that are a significant health burden [112]. For neurodegenerative conditions like Alzheimer's (AD) and Parkinson's (PD) diseases, endo-lysosomes seem to play a critical role [113]. Accordingly, TPC2, in particular, has been linked to both AD [114] and PD [115,116], perhaps a result of aberrant trafficking, and LRKK2 mutation in the case of PD.

TPCs contribute to cardiovascular complications. In blood vessels, TPCs exacerbate hypoxia-induced hypertension [117,118] or macular degeneration [119]. In the heart, the NAADP/TPC axis mediates adrenaline-evoked ionotropy [33] via local Ca^2+^ signals at MCSs [120], and TPC2-KO mice manifest cardiac arrhythmias [121]. Similarly, NAADP/TPCs aggravate ischaemia-reperfusion injury: a regulatory subunit of protein kinase A senses the injury-induced redox changes to switch off the NAADP/TPC-dependent Ca^2+^ release [122] that might otherwise couple to lethal mitochondrial permeability transition [69]; therefore, pharmacological or genetic ablation of NAADP/TPCs protects against ischaemia-reperfusion injury [69,122].

Pathogens deliver toxins and/or enter the host cells to replicate, often gaining access via the endocytic pathway where they traffic through the endo-lysosomal system by co-opting host pathways. Since TPCs regulate vesicular uptake pathways (endocytosis, macropinocytosis, phagocytosis) [41,42,123] and are important for trafficking [86,87,124], their importance in contributing to pathogenicity was likely. Accordingly, inhibiting TPCs reduces infectivity of the Ebola virus [58], and of the Coronaviruses causing MERS [89] and Covid-19 [125,126]. Likewise, reducing the expression of the essential NAADP-binding protein, JPT2, also reduces viral uptake [38]. For HIV-1 replication, the virus subverts TPCs to allow essential Tat protein release [127]. Bacteria require toxins to traffic through the endo-lysosomal system and those for cholera, diphtheria and anthrax rely on TPCs [123,124,128].

An increasing field is that of TPCs in cancer [129] where, remarkably, TPCs impact different aspects. Feeding the tumour requires a blood supply and TPCs help drive angiogenesis [65,104]. Metastatic invasion and migration of the tumour cells themselves is another TPC-dependent process [66,104,130], and finally, tumour proliferation is under TPC2 control [64,66]. Consequently, TPC inhibition reduces tumour mass [64,104], and TPC1 and TPC2 may differentially contribute [131]. It is germane that the NAADP-binding protein, JPT2, is implicated in cancer progression [40].

## Conclusion

In this brief overview, we have highlighted the diversity of both the ionic nature of the TPC signals and the breadth of the (patho)physiological processes in which TPCs play an important role, and this is only set to grow. A common theme is the involvement of TPCs in vesicular trafficking. With still so many unknowns, and the likely intersection with hitherto unsuspected pathways, the field of endo-lysosomal ionic signalling will continue to be a rich source to mine.

## Perspectives

- TPCs are endo-lysosomal Ca^2+^-permeable channels that are emerging as important signal transducers across biology and phyla.
- Unusually, their ion conductances depend on the stimulus: they are plastic channels.
- Different conductances confer the ability of TPCs to signal in different modalities (e.g. Ca^2+^, electrical, lysosomal pH).
- New TPC protein regulators have recently emerged.

## Abbreviations

AD: Alzheimer's diseases
ER: endoplasmic reticulum
GOF: gain-of-function
PD: Parkinson's diseases
TPC: two-pore channel
